# Alleviation of *Porphyromonas gingivalis* or Its Extracellular Vesicles Provoked Periodontitis and Cognitive Impairment by *Lactobacillus pentosus* NK357 and *Bifidobacterium bifidum* NK391

**DOI:** 10.3390/nu15051068

**Published:** 2023-02-21

**Authors:** Xiaoyang Ma, Jong-Wook Yoo, Yoon-Jung Shin, Hee-Seo Park, Young-Hoo Son, Dong-Hyun Kim

**Affiliations:** Neurobiota Research Center, College of Pharmacy, Kyung Hee University, 26, Kyungheedae-ro, Dongdaemun-gu, Seoul 02447, Republic of Korea

**Keywords:** *Porphyromonas gingivalis*, periodontitis, cognitive decline, *Lactobacillus pentosus*, *Bifidobacterium bifidum*, gut microbiota

## Abstract

*Porphyromonas gingivalis* (PG) is closely involved in the outbreak of periodontitis and cognitive impairment (CI). Herein, we examined the effects of anti-inflammatory *Lactobacillus pentosus* NK357 and *Bifidobacterium bifidum* NK391 on PG- or its extracellular vesicles (pEVs)-induced periodontitis and CI in mice. Oral administration of NK357 or NK391 significantly decreased PG-induced tumor necrosis factor (TNF)-α, receptor activator of nuclear factors κB (RANK), and RANK ligand (RANKL) expression, gingipain (GP)^+^lipopolysaccharide (LPS)^+^ and NF-κB^+^CD11c^+^ populations, and PG 16S rDNA level in the periodontal tissue. Their treatments also suppressed PG-induced CI -like behaviors, TNF-α expression and NF-κB-positive immune cells in the hippocampus and colon, while PG-suppressed hippocampal BDNF and *N*-methyl-D-aspartate receptor (NMDAR) expression increased. The combination of NK357 and NK391 additively alleviated PG- or pEVs-induced periodontitis, neuroinflammation, CI-like behaviors, colitis, and gut microbiota dysbiosis and increased PG- or pEVs-suppressed BDNF and NMDAR expression in the hippocampus. In conclusion, NK357 and NK391 may alleviate periodontitis and dementia by regulating NF-κB, RANKL/RANK, and BDNF-NMDAR signaling and gut microbiota.

## 1. Introduction

*Porphyromonas gingivalis* (PG), an opportunistic pathogen, is a major cause of periodontal disease [1]. Patients with periodontitis are often comorbid for dementia including Alzheimer’s disease (AD) [2,3,4]. In particular, moderate and severe periodontitis has reported to be a risk factor of dementia [4]. PG produces lipopolysaccharide (LPS) and gingipains (GPs) as virulence factors [5,6]. Gingivally infected PG or intraperitoneally injected PG LPS impairs cognitive function in mice [6,7,8]. In the brain of dementia patients, GPs are frequently detected. GPs exhibit neurotoxicity in vivo [5,9,10]. Inhibiting GPs alleviates PG-induced neuroinflammation in vivo [9]. 

*Lactobacilli* and *Bifidobacteria*, representative probiotics, have a variety of physiological functions: they mitigate gut microbiota dysbiosis, immune imbalance, inflammation, and cognitive decline [11,12,13]. *Lactobacilus delbrueckii* subsp. bulgaricus regulates the release of Th and Treg cells-involved cytokines in patients with atopic dermatitis [14]. *Lactobacillus acidophilus* improves antibiotics-induced gut dysbiosis and diarrhea [15]. Tumor necrosis factor (TNF)-α expression-suppressing *Lactobacillus mucosae* mitigates cognitive decline in mice [16]. Tight junction protein expression-increasing *Lactococcus lactis* improves 2,4,6-trinitrobenzenesulfonic acid-induced cognitive impairment (CI) and colitis in rodents [17]. Gut microbiota endotoxin production-inhibiting *Bifidobacterium longum* alleviates cognitive function and gut dysbiosis in aged mice [18]. Although probiotics have been reported to be able to alleviate periodontitis and suppress oral pathogen infection in vivo [19,20], the effects of probiotics on gingival pathogen-induced CI have been not studied thoroughly.

Here, we selected anti-inflammatory probiotics *Lactobacillus pentosus* NK357 and *Bifidobacterium bifidum* NK391, which inhibited TNF-α expression in PG-treated macrophages, and investigated their effects on PG-induced periodontitis and CI in mice. 

## 2. Materials and Methods

### 2.1. Materials

Brain heart infusion medium (BHI) was purchased from BD (Franklin Lakes, NJ, USA). Radio immunoprecipitation assay lysis (RIPAL) buffer was purchased from Pierce Biotechnology (Rockford, IL, USA). Millipore-size filters were purchased from Millipore Corp. (Bedford, MA, USA). 

### 2.2. Culture of PG 

PG ATCC 33237 (purchased from American Type Culture Collection, Manassas, VA, USA) was anaerobically cultured in BHI broth containing hemin (5 µg/mL) and menadione (1 µg/mL) (BHIHM) in the anaerobic box [21]. Briefly, PG was anaerobically cultured in BHIHM broth at 37 °C for 2 days and centrifuged (12,000× *g*, 20 min, 4 °C). The collected bacteria were suspended in saline for experimental use.

### 2.3. Preparation of pEVs

pEVs were prepared from the cultured supernatant of PG, as previously reported [22]. The cultured PG was centrifuged (10,000× *g*, 30 min, 4 °C). The supernatant was successively filtrated using 0.45-μm and 0.22-μm millipore filters. The filtrated supernatant was centrifuged (120,000× *g*, 3 h, 4 °C). The precipitate was used as pEVs, which were suspended in saline for experiment use.

### 2.4. Culture of NK357 and NK391

NK357 and NK391 were cultured in general media for probiotics such as MRS broth, centrifuged (5000× *g*, 4 °C, 20 min), and washed with saline. For in vitro experiments, collected probiotics were suspended in saline. For in vivo experiments, collected probiotics were suspended in 1% trehalose.

### 2.5. Culture of SH-SY5Y Cells and BV2 Cells

SH-SY5Y and BV2 cells (purchased from Korea Cell Line Bank, Seoul, Republic of Korea) were cultured at 37 °C in a 95% air and 5% CO_2_ in DMEM containing 1% antibiotic–antimycotic and 5% fetal bovine serum. For assaying cytotoxicity and brain-derived neurotropic factor (BDNF) expression level, SH-SY5Y cells (1 × 10^6^ cells/mL) were incubated with pEVs or heat-killed (90 °C, 10 min) PG (1 × 10^5^ cells/mL) for 24 h. For assaying TNF-α and interleukin (IL)-1β expression levels, BV-2 cells (1 × 10^6^ cells/mL), which are microglial cells derived from C57/BL6 murine, were incubated with pEVs or heat-killed PG for 24 h.

### 2.6. Animals

Male C57BL/6 mice (6-weeks old, 18–21 g) were delivered from Koatech Inc. (Yongin-shi, Republic of Korea). Mice were acclimatized in ventilated controlled standard condition for 7 days before experiment. Mice were fed standard chow diet and water ad libitum. All animal experiments were approved by the Kyung Hee University Institutional Animal Care and Use Committee of (IACUC No., KUASP(SE)-22017). Animal experiments were ethically performed according to the guideline of the University IACU.

For preparation of mice with periodontitis and CI, PG or pEVs were gingivally exposed into mice. Each group consisted of six mice. 

First, we randomly divided mice in five groups (NC, PG, PL, PB, and PM). PG (1 × 10^9^ CFU/mL, in 0.05 mL saline) was infected into the gingiva of four groups (PG, PL, PB, PM) daily for 21 days. Test agents (PG, vehicle; PL, 1 × 10^9^ colony-forming unit (CFU)/mouse of NK357; PB, 1 × 10^9^ CFU/mouse of NK391; PM, 1 × 10^9^ CFU/mouse of NK357 and NK391 (4:1) mix, suspended in 0.1 mL of saline) were orally gavaged daily for 7 days. NC group was treated with vehicle instead of PG and probiotics.

Secondly, we randomly divided mice in three groups (NC, EV, and EM). pEVs (2 μg as protein, in 0.05 mL saline) were exposed into the gingiva of two groups (EV, EM) once a day for 21 days. Test agents (EV, vehicle; EM, 1 × 10^9^ CFU/mouse of NK357 and NK391 [4:1] mix, suspended in 0.1 mL of saline) were orally gavaged for 7 days. NC group was treated with saline instead of pEVs and probiotics.

Cognitive behaviors were measured by the Y-maze task (YMT) and novel object recognition test (NORT) 18 h after the final test agent treatment, as previously reported [18]. Mice were killed in a chamber filled with CO_2_, followed by cervical dislocation 18 h after the final behavioral task. The brain, periodontal, and colon tissues were collected and stored at −80 °C until usage for experiments. 

### 2.7. Determination of Biomarkers

Periodontal, colon, and brain tissues were treated with RIPAL buffer and centrifuged (10,000× *g*, 4 °C, 10 min), as previously reported [23]. In the tissue lysate supernatants and sera, TNF-α, IL-1β, IL-10, prostaglandin E2 (PGE2), and high sensitivity C-reactive protein (hsCRP) levels were assayed using commercial enzyme-linked immunosorbent assay (ELISA) kits. Periodontal alkaline phosphatase activity was measured using Asan alkaline phosphatase activity assay kit (Seoul, Republic of Korea).

### 2.8. Immunofluorescence Assay

Brain, gingiva, and colon tissues were isolated from formaldehyde-perfused mice, post-fixed with p-formaldehyde solution, cyto-protected in sucrose solution, frozen, and sectioned [24]. The sections were blocked with serum, treated with primary antibodies for NF-κB (1:800, Cell Signaling, Danvers, MA, USA), CD11c (1:800, Cell Signaling), LPS (1:500), claudin-5 (1:800, Millipore, Burlington, MA, USA), GP (1:800, Cusabio, Houston, TX, USA), Iba1 (1:1000, Abcam, Cambridge, UK), BDNF (1:800, Abcam), and/or NeuN (1:1000, Abcam) for 15 h, washed twice, and then treated with Alexa Fluor 594- or Alexa Fluor 488 (1:500, Invitrogen)-conjugated secondary antibodies for 2 h. Cell nucleus was stained with 4′,6-diamidino-2-phenylindole. The stained section was scanned using a confocal microscope. 

### 2.9. Microbiota 16S rRNA Sequencing

Bacterial genomes were purified from the fresh periodontal and feces of mice using a QIAamp DNA stool mini kit (Qiagen, Hilden, Germany) and amplified using barcoded primers, which targeted the V4 gene region of bacterial 16S rRNA [16]. The sequencing of amplified genes was carried out using Illumina iSeq 100. Sequenced data were deposited in the NCBI’s short read archive (PRJNA PRJNA895549).

### 2.10. Quantitative Real-Time Polymerase Chain Reaction (qPCR)

For the quantitative analysis of NMDAR, BDNF, and cytokines, mRNAs were extracted from brain and gingiva tissues using a RNeasy Mini Kit and the reverse transcription was performed using a cDNA synthesis kit and oligo dT primer. qPCR was performed using a Qiagen thermal cycler [25]. Thermal cycling was carried out at 95 °C for 30 s, followed by 40–42 cycles of denaturation (95 °C for 5 s) and amplification (70 °C for 30 s). Primer sequences are indicated in Supplementary Table S1. 

### 2.11. Statistical Analysis

Data are indicated as mean ± SD using GraphPad Prism 9. Significance was analyzed using one-way ANOVA followed by Dunnett’s test (*p* < 0.05).

## 3. Results

### 3.1. NK357 and NK391 Decreased PG- or pEVs-Induced TNF-α Expression in BV2 Cells and Increased PG- or pEVs-Suppressed BDNF Expression in SH-SY5Y Cells

**Figure 1:**
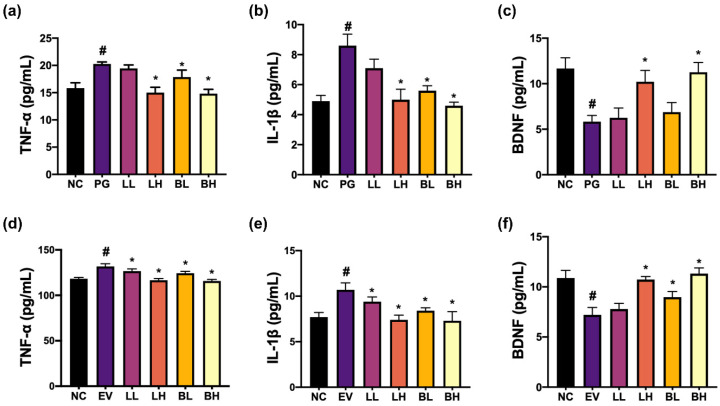
NK357 and NK391 decreased TNF-α and IL-1β expression in PG- or pEVs-stimulated BV2 cells and increased BDNF expression in PG- or pEVs-stimulated SH-SY5Y cells. Effects on PG-induced TNF-α (**a**) and IL-1β expression (**b**) in BV2 cells. (***c***) Effects on PG-suppressed BDNF expression in SH-5Y cells. Effects on pEVs-induced TNF-α (**d**) and IL-1β expression (**e**) in BV2 cells. (**f**) Effects on pEVs-suppressed BDNF expression in SH-SY5Y cells. (**a**–**c**): NC, treated with vehicle; PG, treated with 1 × 10^5^ CFU/mL of PG; LL, 1 × 10^4^ CFU/mL of NK357 with PG; LH, 1 × 10^6^ CFU/mL of NK357 with PG. (**d**–**f**): EV, treated with 50 ng/mL of pEVs; LL, 1 × 10^4^ CFU/mL of NK357 with EV; LH, 1 × 10^6^ CFU/mL of NK357 with EV; BL, 1 × 10^4^ CFU/mL of NK391 with EV; BH, 1 × 10^6^ CFU/mL of NK391 with EV. Data were described as mean ± SD (*n* = 4). # *p* < 0.05 vs. NC. * *p* < 0.05 vs. PG or EV.

To understand whether probiotics could alleviate PG-induced CI with periodontitis in vivo, we first examined the anti-inflammatory activity of probiotics from kimchi and human fecal bacteria collections (Figure 1). Of tested bacteria, NK357, isolated from kimchi, and NK391, isolated from human microbiota, suppressed TNF-α and IL-1β expression in PG- or pEVs-stimulated BV-2 cells Moreover, they increased PG- or pEVs-suppressed BDNF expression in SH-SY5Y cells. Although the combined effect of NK357 with NK391 was not significantly different to that of NK357 or NK391 alone, the effect of the NK351 and NK391 (4:1) mix most potently inhibited TNF-α and IL-1β expression in LPS-stimulated BV2 cells and increased BDNF expression in LPS-stimulated SH-SY5Y cells (Supplement Figure S1). However, they did not show cytotoxicity against these cells at the concentration of 1 *×* 10^9^ CFU/mL. NK357 and NK391 were named *L. pentosus* and *B. bifidum*, respectively, on the basis of gram staining, API kits, and 16S rRNA gene sequences.

### *3.2. NK357 and NK391 Alleviated PG-Induced Periodontitis* in Mice

**Figure 2:**
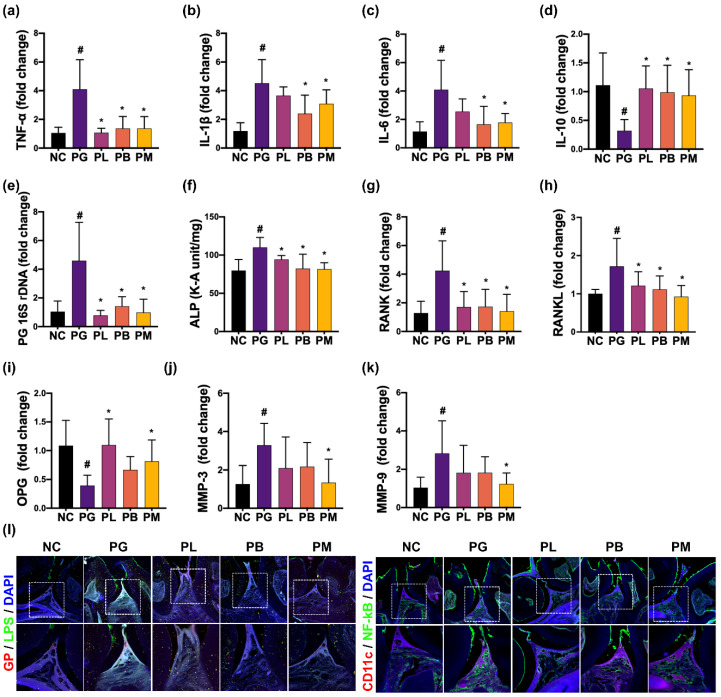
NK357, NK391, and NKc alleviated PG-induced periodontitis in mice. Effects on the expression of TNF-α (**a**), IL-1β (**b**), IL-6 (**c**), and IL-10 (**d**) and PG 16S rDNA level (**e**) in the periodontal tissue. Effects on the expression of alkaline phosphatase activity (**f**) and RANK (**g**), RANKL (**h**), OPG (**i**), MMP-3 (**j**), and MMP-9 expression (**k**) and GP^+^LPS^+^ and NF-κB^+^CD11c^+^ cell populations (**l**) in the periodontal tissue. Data were described as mean ± SD (*n* = 6). # *p* < 0.05 vs. NC. * *p* < 0.05 vs. PG.

Next, the effects of NK357 and NK391 on PG-induced periodontitis were investigated in mice (Figure 2). When PG was orally exposed to mouth for 3 weeks, exposure to PG increased IL-1β, IL-6, along with TNF-α expression and GP^+^LPS^+^ and NF-κB^+^CD11c^+^ cell populations in the periodontal tissue, while IL-10 expression was decreased. Moreover, PG 16S rRNA gene was found in the periodontal tissue of PG-exposed mice. However, oral administration of NK357 or NK391 down-regulated PG-increased IL-1β, IL-6, along with TNF-α expression and GP^+^LPS^+^ and NF-κB^+^CD11c^+^ cell populations and increased PG-decreased IL-10 expression. PG 16S rDNA level was also decreased. The combination of NK357 with NK391 (NKc) additively decreased PG 16S rDNA levels as well as suppressed PG-induced periodontitis: they suppressed TNF-α and IL-6 expression and GP^+^LPS^+^ and NF-κB^+^CD11c cell populations in the periodontal tissue. We also investigated the effects of NK357 and NK391 on PG-induced periodontitis in mice (Figure 2). When PG was orally exposed for 3 weeks, PG increased IL-1β, IL-6, TNF-α, RANK, RANKL, MMP-3, and MMP-9 expression and GP^+^LPS^+^ and NF-κB^+^CD11c^+^ cell populations in the periodontal tissue, while IL-10 expression was reduced. Moreover, PG 16S rRNA gene was detected in the periodontal tissue of PG-exposed mice. However, oral gavage of NK357 or NK391 significantly down-regulated PG-induced TNF-α, IL-1β, IL-6, RANKL, and RANK expression, alkaline phosphatase activity, and GP^+^LPS^+^ and NF-κB^+^CD11c^+^ cell populations and up-regulated PG-suppressed IL-10 and OPG expression. PG 16S rRNA gene level was also reduced. The combination of NK357 with NK391 (NKc) additively decreased PG 16S rDNA levels and suppressed PG-induced periodontitis: the combination suppressed IL-6 and TNF-α expression and GP^+^LPS^+^ and NF-κB^+^CD11c^+^ cell populations in the periodontal tissue. NK357, NK391, and NKc weakly, but not significantly, inhibited the growth of PG in in vitro study.

### *3.3. NK357 and NK391 Alleviated PG-Induced CI* in Mice

**Figure 3:**
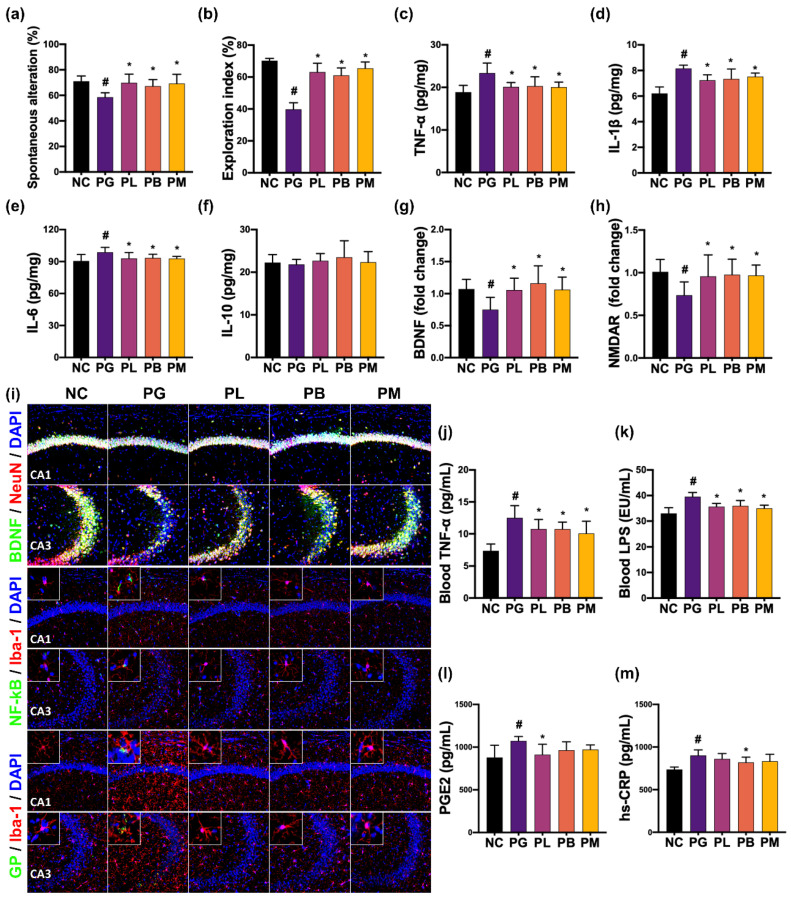
NK357 and NK391 alleviated PG-induced CI in mice. Effects on spontaneous alternation (**a**) and exploration (**b**). Effects on hippocampal TNF-α (**c**), IL-1β (**d**), IL-6 (**e**), and IL-10 (**f**), BDNF (**g**), and NMDAR expression (**h**). (**i**) Effects on hippocampal BDNF^+^NeuN^+^ cells, NF-κB^+^Iba1^+^, and GP^+^Iba1^+^ cell populations. Effects on blood TNF-α (**j**), LPS (**k**), PGE2 (**l**), and hs-CRP (**m**) levels. Data are indicated as mean ± SD (*n* = 6). # *p* < 0.05 vs. NC. * *p* < 0.05 vs. PG.

The effects of NK357 and NK391 on gingivally exposed PG-induced CI were investigated in mice (Figure 3). Gingivally exposed PG also significantly impaired cognitive behaviors in the YMT and NORT to 75.2% (F_4,25_ = 4.98, *p* = 0.004) and 73.5% (F_4,25_ = 48.09, *p* < 0.001) of NC group, respectively. Gingivally infected PG increased IL-1β, IL-6, and TNF-α expression and GP^+^Iba1^+^ and NF-κB^+^Iba1^+^ cell populations in the hippocampus, while BDNF^+^NeuN^+^ cell population decreased. IL-10 expression did not fluctuate after treatment with PG. However, oral administration of NK357, NK391, or NKc significantly decreased PG-induced CI-like behaviors in the YMT to 98.4%, 94.6%, and 97.5% (F_4,25_ = 4.98, *p* = 0.004) of NC group, respectively, and NORT to 89.9%, 86.9%, and 93.2% (F_4,25_ = 48.09, *p* < 0.001) of NC group, respectively. However, PG treatment did not affect general locomotor activity, as previously reported [26]. Treatment with NK357, NK391, or NKc decreased PG-induced hippocampal expression of IL-1β, IL-6, and TNF-α and populations of NF-κB^+^Iba1^+^ and GP^+^Iba1^+^ cells, while PG-suppressed BDNF, and NMDAR expression and BDNF^+^NeuN^+^ cell population increased. Their treatments did not affect hippocampal IL-10 expression. However, their treatments lowered TNF-α, LPS, hs-CRP, and PGE2 levels in the blood. 

### *3.4. NK357 and NK391 Alleviated PG-Induced Colitis and Gut Dysbiosis* in Mice

**Figure 4:**
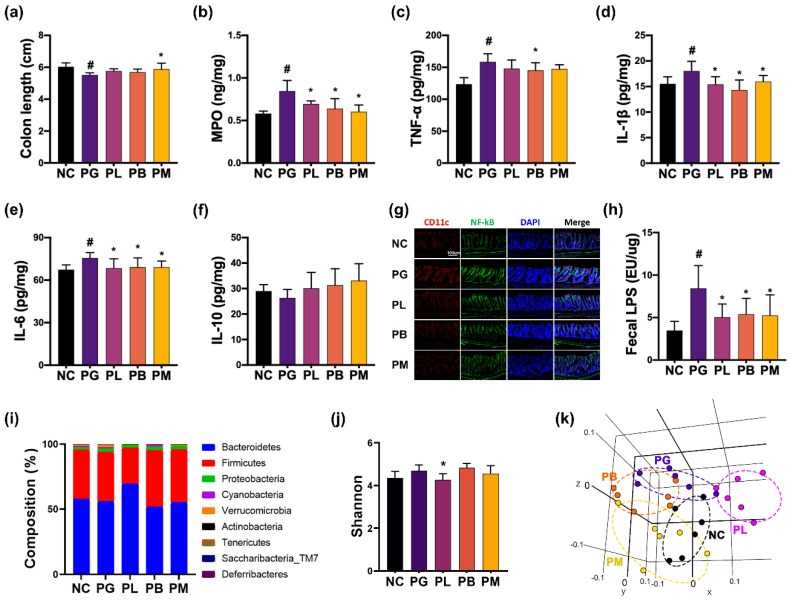
NK357, NK391 and NKc alleviated PG-induced colitis and gut microbiota dysbiosis in mice. Effects on colon length (**a**), MPO (**b**), TNF-α (**c**), IL-1β (**d**), IL-6 (**e**), and IL-10 expression (**f**), and NF-κB^+^CD11c^+^ cell population (**g**). (**h**) Effects on fecal LPS level. Effects on gut bacterial composition: (**i**) phylum, (**j**) Shannon index (α-diversity), and (**k**) β-diversity (principal coordinate analysis plot based on UniFrac). Data were indicated as mean ± SD (*n* = 6). # *p* < 0.05 vs. NC. * *p* < 0.05 vs. PG.

The effects of NK357, NK391, and NKc on PG-induced colitis were investigated in mice (Figure 4). Gingivally exposed PG caused colon shortening and increased the expression of myeloperoxidase (MPO), IL-1β, IL-6, and TNF-α and population of NF-κB^+^CD11c^+^ cells in the colon, while IL-10 expression was not significantly affected. However, oral administration of NK357, NK391, or NKc partially restored PG-induced colon shortening and MPO, IL-1β, and IL-6 expression, along with NF-κB^+^CD11c^+^ cell population, to those of the NC group.

Gingivally exposed PG altered gut microbiota composition, assessed by 16S rDNA pyrosequencing (Figure 4, Supplement Tables S2–S5). Exposure to PG shifted the β-diversity of gut microbiota, while α-diversity, significantly, did not fluctuate. *PG* exposure increased *Akkermansiaceae* population, while *Lactobacillaceae* and *Enterococcaceae* populations decreased. However, oral administration of NK357, NK391, or NKc decreased *Verrucomicrobia*, *Erysipelorichaceae*, and *Akkermansiaceae* populations. In particular, NK357 treatment increased *Muribaculaceae* population and decreased *Ruminococcaceae* population. They also decreased PG-induced fecal LPS level.

### *3.5. NKc Alleviated pEVs-Induced Periodontitis, CI, and Colitis* in Mice

**Figure 5:**
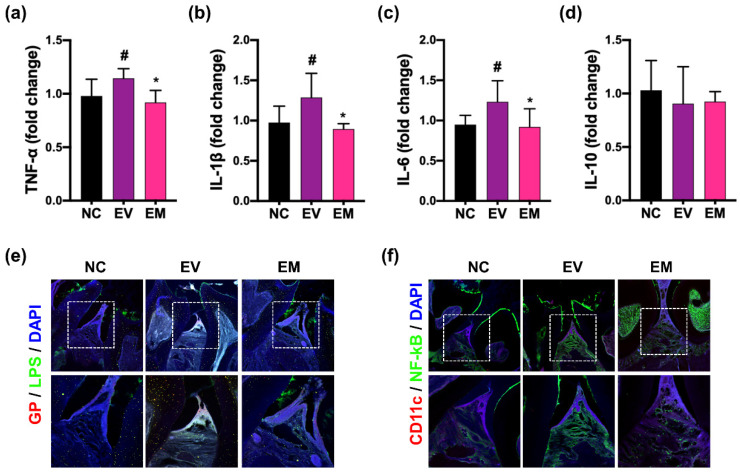
NKc alleviated pEVs-induced periodontitis in mice. Effect on TNF-α (**a**), IL-1β (**b**), IL-6 (**c**), and IL-10 expression (**d**) in the periodontal tissue. Effect on GP^+^LPS^+^ (**e**) and NF-κB^+^CD11c^+^ cell populations (**f**) in the periodontal tissue. Data were indicated as mean ± SD (*n* = 6). # *p* < 0.05 vs. NC. * *p* < 0.05 vs. EV.

NKc, the combination of NK357 with NK391, additively PG-induced periodontitis, CI, and colitis in mice. Therefore, to understand the efficacy of NKc on periodontitis, CI, and colitis, the effect of NKc on pEVs-induced periodontitis, CI, and colitis was investigated in mice (Figure 5). First, gingivally exposed pEVs increased TNF-α, IL-1β, and IL-6 expression, and GP^+^LPS^+^ and NF-κB^+^CD11c^+^ cell populations in the periodontal tissue, while IL-10 was not affected. However, oral administration of NKc significantly decreased pEVs-induced IL-1β, IL-6, and TNF-α expression and GP- and NF-κB-positive cell populations in the periodontal tissue. 

**Figure 6:**
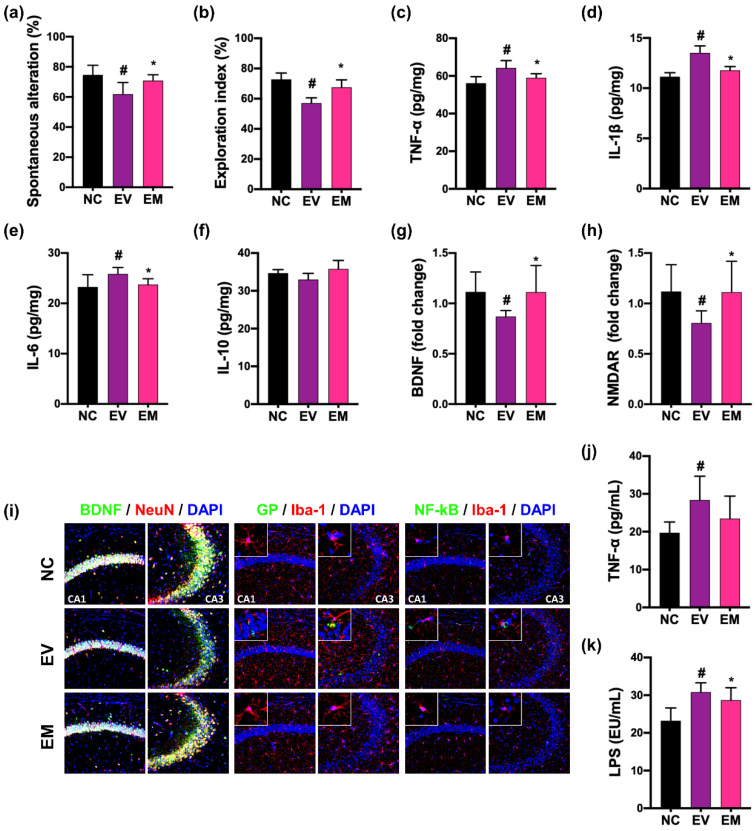
NKc alleviated pEVs-induced CI in mice. Effect on spontaneous alternation (**a**) and exploration (**b**). Effect on the hippocampal expression of TNF-α (**c**), IL-1β (**d**), IL-6 (**e**), IL-10 (**f**), BDNF (**g**), and NMDAR (**h**) and populations of BDNF^+^NeuN^+^ cells, GP^+^Iba1^+^, and NF-κB^+^Iba1^+^ cells (**i**). Effect on blood TNF-α (**j**) and LPS levels (**k**). Data were described as mean ± SD (*n* = 6). # *p* < 0.05 vs. NC. * *p* < 0.05 vs. EV.

Gingivally exposed pEVs also increased CI-like behaviors in mice in the YMT and NORT to78.4% (F_2,15_ = 6.77, *p* = 0.008) and 72.7% (F_2,15_ = 21.52, *p* < 0.001) of NC group, respectively (Figure 6). pEVs treatment also increased the hippocampal expression of IL-1β, IL-6, and TNF-α and populations of GP^+^Iba1^+^ and NF-κB^+^Iba1^+^ cells, while BDNF^+^NeuN^+^ cell population decreased. Oral administration of NKc significantly alleviated pEVs-induced CI-like behaviors in the YMT and NORT to 94.8% (F_2,15_ = 6.77, *p* = 0.008) and 92.7% (F_2,15_ = 21.52, *p* < 0.001) of NC group, respectively. NKc also down-regulated pEVs-induced hippocampal expression of IL-1β, IL-6, and TNF-α and population of NF-κB^+^Iba1^+^ and GP^+^Iba1^+^ cells, while pEVs-suppressed BDNF, and NMDAR expression and BDNF^+^NeuN^+^ cell population was upregulated. Their treatments decreased TNF-α and LPS levels in the blood.

**Figure 7:**
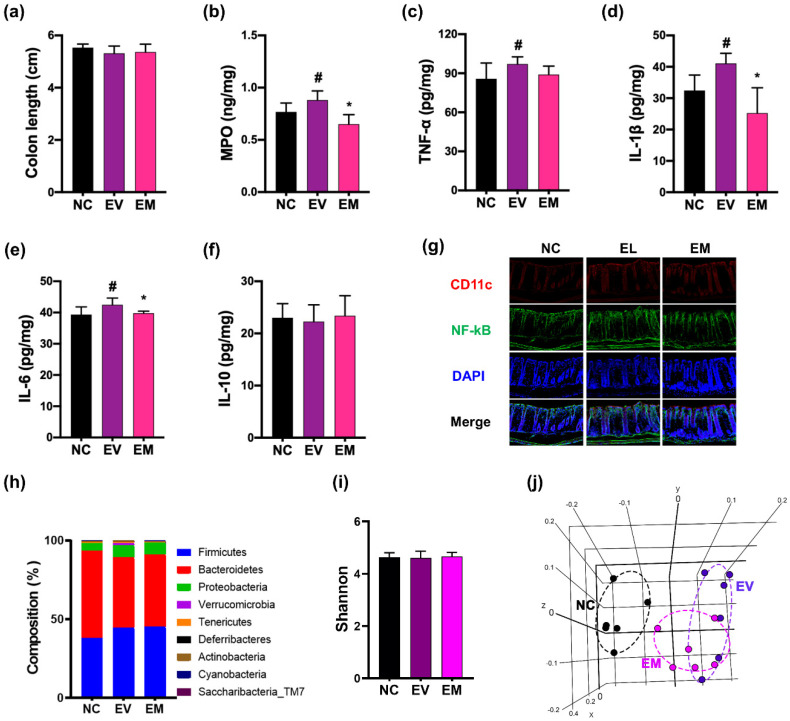
NKc alleviated pEVs-induced colitis and gut microbiota dysbiosis in mice. Effect on colon length (**a**), expression of MPO (**b**), TNF-α (**c**), IL-1β (**d**), IL-6 (**e**), and IL-10 (**f**), and population of NF-κB^+^CD11c^+^ cells (**g**). Effect on gut bacterial composition: (**h**) phylum, (**i**) Shannon index (α-diversity), and (**j**) β-diversity (based on UniFrac). Data were described as mean ± SD (*n* = 6). # *p* < 0.05 vs. NC. * *p* < 0.05 vs. EV.

Gingivally exposed pEVs up-regulated the colonic expression of MPO, IL-1β, and IL-6 and population of NF-κB^+^CD11c^+^ cells (Figure 7). Oral administration of NKc partially restored pEVs-induced expression of MPO, IL-1β, and IL-6 and population of NF-κB^+^CD11c^+^ cells.

Gingivally exposed pEVs also altered gut microbiota composition (Figure 7, Supplement Tables S6–S9). Exposure to pEVs shifted the β-diversities of gut microbiota, while α-diversity did not fluctuate. pEVs treatment decreased *Prevotellaceae* and FR888536_f (belonging to Cyanobacteria) populations, while *Verrucomicrobia* and *Helicobacteriaceae* populations increased. However, oral administration of NKc weakly increased *Prevotellaceae* and FR888536_f and decreased *Verrucomicrobia* population. 

## 4. Discussion

Gut microbiota dysbiosis, which is caused by exposed stressors such as antibiotics and pathogens, is closely associated with dementia [27,28]. The infection of pathogens into the intestine causes gastrointestinal inflammation, which increases the translocation of gut bacterial byproducts such as endotoxins and exotoxins into the body through increased gastrointestinal permeability [29,30,31]. Exposure to immobilization stress cause microbiota dysbiosis, including *Escherichia coli* overgrowth and LPS overexpression, and anxiety/depression [32]. Furthermore, *E. coli* or bacterial LPS causes colitis and CI in mice [16,33]. Oral infection of *PG* or intraperitoneal exposure to its LPS causes neuroinflammation and neurotoxicity in mice [7,8]. pEVs also shows neurotoxicity in vivo [6,34]. The results suggest that the induction of gastrointestinal inflammation including periodontitis and colitis by the infection of pathogens can cause psychiatric disorders.

In the present study, gingivally infected PG or pEVs increased GP^+^LPS^+^ and NF-κB^+^CD11c^+^ cell populations, TNF-α, IL-1β, RANK, and RANKL expression, and alkaline phosphatase activity in the periodontal tissue and NF-κB^+^Iba1^+^ and GP^+^Iba1^+^ cell populations and proinflammatory cytokine expression in the hippocampus. However, exposure to PG or pEVs suppressed OPG in the periodontal tissue. These findings suggest that gingivally exposed PG or pEVs may cause periodontitis, periodontal bone loss, and neuroinflammation in vivo by suppressing NF-κB and RANK/RANKL signaling pathways. 

Exposure to PG or pEVs increased CI-like behaviors in mice. They decreased BDNF^+^NeuN^+^ cell population and BDNF and NMDAR expression in the brain. Gut microbiota regulates BDNF expression in the central nerve system, which modulates NMDAR production [35,36]. NF-κB activation suppresses BDNF expression in the brain [37,38]. These findings imply that PG and pEVs can impair cognitive function by suppressed NF-κB-involved BDNF/NMDAR expression. Furthermore, gingivally exposed PG increased TNF-α, hsCRP, PGE2, and LPS levels in the blood. Moreover, gingivally exposed PG or pEVs caused colitis and altered the composition of gut microbiota: they shifted β-diversity, increased *Akkermansiaceae* (belonging to *Verrucomicrobia*) population, and decreased *Lactobacillacease* population. These findings imply that PG can produce pEVs in the gingiva and PG and pEVs cause periodontitis, which may accelerate the translocation of PG byproducts such as pEVs and LPS into the brain, resulting in neuroinflammation and CI.

Orally administered NK357 and NK391 mitigated *PG*-induced periodontitis and CI-like behaviors in mice. They also suppressed PG-induced expression of TNF-α and IL-1β and population of NF-κB-activated cells in the periodontium and brain. Furthermore, they alleviated PG-induced RANK, RANKL, MMP-3, and MMP-9 expression and PG-suppressed OPG expression in the periodontal tissue. They reduced PG 16S rDNA level and GP^+^LPS^+^ cell population in the periodontal tissue and GP^+^Iba1^+^ cell populations in the brain. They reduced PG-induced blood LPS and TNF-α levels. Gatej et al. reported that *Lactobacillus rhamnosus* GG prevents PG/*Fusobacterium nucleatum*-induced periodontitis and alveolar bone loss in mice [19]. Kim et al. reported that *Weissella cibaria* CMU alleviated ligature-induced periodontitis in mice [39]. Maorales et al. reported that orally administered *L. rhamnosus* SP1 improved periodontitis in volunteers [20]. These findings imply that NK357 and NK391 can improve PG- and pEVs-induced periodontitis and neuroinflammation by regulating immune cells rather than directly inhibiting PG growth.

Oral administration of NK357 or NK391 increased PG-suppressed BDNF and NMDAR expression in the brain. They down-regulated PG- or pEVs-induced GP^+^NF-κB^+^ cell population in the brain. They also down-regulated PG-induced expression of TNF-α and IL-6 and populations of NF-κB-positive cells in the colon. They also improved PG-induced gut microbiota fluctuation: they suppressed *Verrucomicrobia* population and increased *Prevotellaceae* population and LPS production. *L. mucosae* NK41 mitigated *E. coli*- or LPS-induced CI, colitis, and gut microbiota fluctuation by inhibiting NF-κB activation and inducing BDNF expression [16]. *B. longum* NK46 improves cognitive function, colitis, and gut dysbiosis in 5xFAD transgenic mice [18]. These findings imply that NK357 and NK391can alleviate oral pathogen-induced periodontitis, colitis, and CI by inducing NF-κB-involved BDNF and NMDAR expression through the regulation of gut microbiota and their byproducts. 

NKc (the mix of NK357 and NK391) additively alleviated PG- or pEVs-induced periodontitis, neuroinflammation, and colitis in vivo. In particular, NKc strongly restored PG- or pEV-suppressed BDNF and NMDAR expression and BDNF^+^NeuN^+^ cell population in the brain. NKc also down-regulated PG- or pEV-increased TNF-α and IL-1β expression in the periodontal tissue, blood, and brain. These findings imply that NK357 and NK391can additively alleviate gingival pathogen-induced periodontitis, colitis, neuroinflammation, and CI by suppressing NF-κB and RANK/RANKL signaling pathways and modulating gut microbiota. 

## 5. Conclusions

Gingival pathogens such as PG can cause periodontitis by activating NF-κB and RANK/RANKL signaling pathways, resulting in neuroinflammation, colitis, dysbiosis, and CI. NK357 and NK391 can alleviate gingival pathogen-induced periodontitis and systemic inflammation by suppressing NF-κB and RANK/RANKL signaling pathways and modulating gut microbiota, leading to the attenuation of CI. They additively display their effects, thereby being beneficial for the therapy of periodontitis and dementia.

## Data Availability

The datasets used and/or analyzed during the current study are available from the corresponding author on reasonable request.

## Supplementary Materials

The following supporting information can be downloaded at: https://www.mdpi.com/article/10.3390/nu15051068/s1, Table S1. Primers used for qPCR in the study. Table S2. Effects of NK357 and NK391 on the gut microbiota composition at the phylum level in mice with PG-induced periodontitis and CI. Table S3. Effects of NK357 and NK391 on the gut microbiota composition at the family level in mice with PG-induced periodontitis and CI. Table S4. Effects of NK357 and NK391 on the gut microbiota composition at the genus level in mice with PG-induced periodontitis and CI. Table S5. Effects of NK357 and NK391 on the gut microbiota composition at the species level in mice with PG-induced periodontitis and CI. Table S6. Effect of NKc on the gut microbiota composition at the phylum level in mice with pEVs-induced periodontitis and CI. Table S7. Effects of NKc on the gut microbiota composition at the family level in mice with pEVs-induced periodontitis and CI. Table S8. Effects of NKc on the gut microbiota composition at the genus level in mice with pEVs-induced periodontitis and CI. Table S9. Effects of NKc on the gut microbiota composition at the species level in mice with pEVs-induced periodontitis and CI. Figure S1. The combined effects of NK357 and NK391 on the TNF-α and IL-1β expression in PG-stimulated BV2 cells and increased BDNF expression in PG-stimulated SH-SY5Y cells.
