# Supplementary material for: Alleviation of Porphyromonas gingivalis or Its Extracellular Vesicles Provoked Periodontitis and Cognitive Impairment by Lactobacillus pentosus NK357 and Bifidobacterium bifidum NK391
Source: Nutrients. 2023 Feb 21;15(5):1068. doi: 10.3390/nu15051068 (PMC10005711; doi:10.3390/nu15051068)
Supplement: Supplementary file 1 [file nutrients-15-01068-s001.zip › nutrients-2235670-supplementary.pdf]

[Supplement]

Alleviation of *Porphyromonas gingivalis*-provoked periodontitis and cognitive impairment by *Lactobacillus pentosus* NK357 and *Bifidobacterium bifidum* NK391

Table S1. Primers used for qPCR in the present study

Gene	Primer	
	Forward	Reverse
TNF-α	5′-AGCCCACGTAGCAAACCACCAA-3′	5′-ACA5′-GTGCAAGTGACTCAGGGTGA-3′
IL-1β	5′- GCTGAAGGAGTT-GCCAGAAA-3′	5’-CCCATTCCCTTCACAGAGCAAT-3′
IL-6	5′- TAG-TCCTTCCTACCCCAATTTCC-3′	5′-TTGGTCCTTAGCCACTCCTTC-3′
IL-10	5′-CAGCCGGGAAGACAATAACTG-3′	5′-CCGCAGCTCTAGGAGCATGT-3′
BDNF	5′-AGCTGAGCGTGTGTACAGT-3’	5′-TCCATAGTAAGGGCCCGAAC-3′
NMDAR	5′-ATTCATGCAGCCCTTTCAGA-3′	5′-CCTTCCCCAATGCCAGAGT-3′
OPG	5′-AGCCATTGCACACCTCAC-3′	5′-CGTGGTACCAAGAGGACAGAGT-3′
RANK	5′-ATCTCGGACGGTGTTGCAG-3′	5′-TCTTCATTCCAGGTGTCCAAGTA-3′
RANKL	5′-TCCTAACAGAATATCAGAAGACAG-3′	5′-AGGCTTGCCTCGCTGGGCCACATC-3′
MMP-3	5′-CCTGCTTTGTCCTTTGATGC-3′	5′-TGAGTCAATCCCTGGAAAGTC-3′
MMP-9	5′-ATCCAGTTTGGTGTCGCGGAGC-3′	5′-GAAGGGGAAGACGCACAGCT-3′
GAPDH	5′-TGCAGTGGCAAAGTGGAGAT-3′	5′-TTTGCCGTGAGTGGAGTCATA-3′

Table S2. Effects of NK357 and NK391 on the gut microbiota composition at the phylum level in mice with *Porphyromonas gingivalis*-induced periodontitis and cognitive impairment

Taxon Name	Composition (%)				
	NC	PG	LP	BB	Mix
Bacteroidetes	57.8±12.5	56.0±13.1	69.4±9.7	51.8±16.1	55.3±15.3
Firmicutes	37.8±13.5	37.8±11.9	27.9±9.4	43.3±16.0	40.6±15.4
Proteobacteria	1.9±0.7	2.6±0.7	2.1±0.9	3.0±1.1	2.7±0.4
Cyanobacteria	1.0±1.5	1.1±1.2	0.1±0.1	1.0±1.0	0.2±0.2
Verrucomicrobia	0.9±0.4	1.8±2.2	0.0±0.0	0.3±0.3	0.7±0.4
Actinobacteria	0.3±0.2	0.3±0.2	0.3±0.1	0.3±0.1	0.2±0.1
Tenericutes	0.2±0.2	0.3±0.2	0.2±0.2	0.1±0.1	0.2±0.2
Saccharibacteria_TM7	0.0±0.0	0.1±0.1	0.0±0.0	0.0±0.0	0.0±0.0
Deferribacteres	0.0±0.0	0.1±0.1	0.0±0.0	0.2±0.2	0.1±0.0

Values indicate means±SD. $^{\#}p < 0.05$ vs. NC. $^{*}p<0.05$ vs. PG.

Table S3. Effects of NK357 and NK391 on the gut microbiota composition at the family level in mice with *Porphyromonas gingivalis*-induced periodontitis and cognitive impairment

Taxon Name	Composition (%)				
	NC	PG	LP	BB	Mix
Muribaculaceae	36.5±5.6	35.7±12.1	50.6±8.1*	32.2±12.3	31.1±7.4
Lactobacillaceae	15.1±7.2	7.8±4.0	9.5±9.3	3.7±2.4	5.3±3.4

Prevotellaceae	15.0±8.4	13.9±7.6	9.4±1.8	14.1±4.7	18.9±8.8
Lachnospiraceae	13.7±10.2	19.5±11.5	13.7±7.9	29.3±16.6	25.4±15.1
Ruminococcaceae	6.1±2.9	6.7±1.9	4.0±1.3*	8.9±1.7	7.6±1.3
Rikenellaceae	3.0±1.5	3.4±0.8	4.2±3.2	2.3±0.3*	1.6±0.3
Erysipelotrichaceae	2.2±2.2	2.3±1.1	0.1±0.1*	0.6±0.5*	1.8±2.3
Bacteroidaceae	1.9±1.0	1.5±0.6	2.5±1.1	1.8±1.3	3.0±2.4
FR888536_f	1.0±1.5	1.1±1.2	0.1±0.1	1.0±1.0	0.2±0.2
Akkermansiaceae	0.9±0.4	1.8±2.2	0.0±0.0	0.3±0.3	0.7±0.4
AC160630_f	0.7±0.5	0.7±0.4	1.4±0.9	0.7±0.5	0.1±0.1*
Desulfovibrionaceae	0.7±0.6	1.0±0.6	0.9±0.5	1.1±0.5	0.9±0.5
Porphyromonadaceae	0.4±0.2	0.5±0.2	0.4±0.2	0.3±0.2	0.5±0.3
Helicobacteraceae	0.4±.2	0.7±0.4	0.7±0.5	1.4±1.0	0.9±0.5
Sutterellaceae	0.3±0.2	0.3±0.2	0.2±0.1	0.3±0.2	0.6±0.4
Coriobacteriaceae	0.3±0.2	0.3±0.2	0.3±0.1	0.2±0.1	0.1±0.1
Rhodospirillaceae	0.2±0.3	0.4±0.6	0.2±0.2	0.2±0.1	0.2±0.3
Enterococcaceae	0.2±0.1	0.1±0.0#	0.1±0.1	0.1±0.0*	0.1±0.1
Christensenellaceae	0.2±.1	0.3±0.1	0.2±0.1*	0.3±0.1	0.1±0.1*
Odoribacteraceae	0.1±0.1	0.2±0.1	0.8±0.7	0.3±0.2	0.2±0.1
Enterobacteriaceae	0.1±0.0	0.1±0.1	0.0±0.0	0.0±0.0	0.0±0.1
PAC000197_f	0.1±0.1	0.2±.2	0.1±0.1	0.1±0.1	0.1±.1
PAC001057_f	0.1±0.1	0.1±0.1	0.1±0.1	0.0±0.0	0.1±0.1
Streptococcaceae	0.1±0.0	0.1±0.0	0.1±0.0	0.1±0.0	0.1±0.0
Dehalobacterium_f	0.1±0.1	0.1±0.0	0.1±0.1	0.2±0.2	0.1±0.1

Values indicate means±SD. #p < 0.05 vs. NC. *p<0.05 vs. PG.

Table S4. Effects of NK357 and NK391 on the gut microbiota composition at the genus level in mice with *Porphyromonas gingivalis*-induced periodontitis and cognitive impairment

Taxon Name	Composition (%)				
	NC	PG	LP	BB	Mix
Lactobacillus	15.1±7.2	7.7±4.0	9.5±9.2	3.7±2.4	5.3±3.4
PAC001068_g	14.5±2.1	11.0±4.5	16.1±2.9*	13.1±5.2	9.2±2.9
PAC001112_g	5.7±2.6	4.7±1.8	2.8±1.0*	3.3±1.7	2.6±0.3*
Prevotella	5.4±3.9	6.0±4.6	0.8±0.5*	6.0±2.1	8.1±4.4
Paraprevotella	5.0±2.0	0.7±1.3#	0.6±0.7	2.2±2.0	6.5±3.5*
Prevotellaceae_uc	4.6±4.3	7.1±4.0	8.0±2.7	6.0±3.6	4.2±2.2
Muribaculum	3.2±0.5	2.5±1.8	4.9±1.8*	3.3±1.4	3.6±0.8
PAC000186_g	3.1±0.9	4.8±4.0	14.9±4.2*	2.2±1.3	7.7±3.2
PAC000198_g	2.7±0.6	4.9±1.2#	4.4±1.6	2.6±1.0 *	3.1±0.8 *
Alistipes	2.6±1.4	2.6±1.0	3.5±3.0	1.7±0.3	1.0±0.3 *
Faecalibaculum	2.1±2.1	0.5±0.1	0.1±0.1 *	0.3±0.3	1.7±2.3
Ruminococcus	1.9±1.8	2.2±0.9	1.0±0.9	2.7±1.3	2.0±0.8
Bacteroides	1.9±1.0	1.5±0.6	2.5±1.1	1.8±1.3	3.0±2.4
PAC000664_g	1.9±1.5	3.6±3.6	2.0±1.6	2.4±1.6	4.4±2.8

PAC002400_g	1.6±0.3	0.9±0.6#	0.6±0.3	1.1±0.6	0.5±0.3
PAC001127_g	1.5±0.4	0.9±0.5	1.9±1.4	0.8±0.2	0.6±0.2
KE159538_g	1.5±1.3	0.7±0.4	1.3±1.8	3.5±2.5*	2.1±2.0
PAC000661_g	1.1±0.7	0.9±0.4	0.5±0.2*	1.2±0.8	1.2±1.5
FR888536_g	1.0±1.5	1.1±1.2	0.1±0.1	1.0±1.0	0.2±0.2
Pseudoflavonifractor	0.9±0.7	1.2±0.4	0.9±0.4	1.7±1.0	1.4±0.9
PAC001066_g	0.9±0.8	0.8±0.3	1.8±0.8*	1.1±0.4	0.7±0.3
Akkermansia	0.9±0.4	1.8±2.2	0.0±0.0	0.3±0.3	0.7±0.4
PAC001092_g	0.9±0.9	0.5±0.2	0.7±0.8	1.4±1.0	1.1±0.9
PAC001472_g	0.8±0.2	0.7±0.5	0.5±0.3	0.9±0.4	0.8±0.4
PAC002482_g	0.7±0.5	0.7±0.4	1.4±0.9	0.7±0.5	0.1±0.1

Values indicate means±SD.

Table S5. Effects of NK357 and NK391 on the gut microbiota composition at the species level in mice with *Porphyromonas gingivalis*-induced periodontitis and cognitive impairment

Taxon Name	Composition (%)				
	NC	PG	LP	BB	Mix
Lactobacillus murinus group	9.8±6.0	4.5±2.9	4.6±7.0	2.1±1.9	2.8±2.3
PAC001070_s group	7.5±2.5	5.0±3.7	9.1±2.0*	6.1±2.5	2.3±1.7
FJ880724_s	5.0±2.0	0.7±1.3#	0.6±0.7	2.2±2.0	6.5±3.5*
AB702765_s	4.4±3.4	5.4±4.1	0.3±0.5*	5.2±1.7	6.4±3.7
PAC001112_s	3.6±2.3	2.8±1.7	0.1±0.1*	1.2±0.7	1.3±0.3
PAC001072_s	3.5±1.1	1.8±0.7#	2.3±0.9	2.0±0.7	2.1±0.8
Lactobacillus reuteri group	3.2±2.7	2.0±1.2	2.4±1.3	0.8±0.5	1.0±0.6
Faecalibaculum rodentium	2.1±2.1	0.5±0.1	0.1±0.1*	0.3±0.3	1.7±2.3
Muribaculum intestinale	1.8±0.4	1.1±0.6#	1.4±0.4	0.8±0.2	1.6±0.7
PAC002400_s	1.6±0.3	0.9±0.6#	0.6±0.3	1.1±0.6	0.5±0.3
Lactobacillus gasseri group	1.6±1.5	1.1±0.9	1.8±1.1	0.5±0.4	1.2±0.7
PAC001064_s	1.5±0.6	1.2±0.8	1.4±1.5	0.7±0.4	2.9±1.3
PAC002399_s	1.5±0.4	0.9±0.5	1.9±1.4	0.8±0.2	0.6±0.2
Ruminococcus_uc	1.2±2.1	0.0±0.0	0.6±0.9	0.0±0.0*	0.0±0.0
PAC001075_s	1.2±0.3	2.0±0.6#	1.4±0.4	1.2±0.6	1.3±0.4*
AB599946_s	1.1±0.8	0.9±0.4	1.8±0.9	0.8±0.6	0.9±0.4
PAC001084_s	1.0±0.3	0.5±0.1#	1.3±0.4*	1.3±0.5*	1.2±0.2*
Alistipes finegoldii	0.9±0.8	0.6±0.3	1.4±1.2	0.5±0.4	0.2±0.1*
PAC002447_s	0.9±0.3	2.2±2.9	11.4±2.2*	0.1±0.1	3.8±2.1
PAC001066_s	0.9±0.8	0.8±0.3	1.8±0.8*	1.1±0.4	0.7±0.3
Akkermansia muciniphila	0.9±0.4	1.8±2.2	0.0±0.0	0.3±0.3	0.7±0.4
PAC001076_s	0.8±0.6	0.2±0.1#	0.3±0.3	0.7±1.1	0.8±0.3*

EU504031_s group	0.8±0.2	0.7±0.5	0.5±0.3	0.8±0.3	0.8±0.4
EF097112_s	0.8±0.5	1.0±0.2	0.8±0.3	1.3±0.9	1.3±0.4
PAC001097_s	0.7±0.9	0.3±0.2	1.2±1.4	0.3±0.5	1.2±1.4

Values indicate means±SD. $^{\#}p < 0.05$ vs. NC. $^{*}p<0.05$ vs. PG.

Table S6. Effects of NKc on the gut microbiota composition at the phylum level in mice with pEVs-induced periodontitis and cognitive impairment

Taxon Name	Composition (%)		
	NC	EV	Mix
Firmicutes	38.0±5.4	44.8±9.1	45.2±2.8
Bacteroidetes	55.4±7.2	44.4±8.4#	46.0±4.4
Proteobacteria	5.0±2.4	7.9±2.3	7.4±3.6
Verrucomicrobia	0.0±0.0	1.3±2.1	0.1±0.1
Tenericutes	1.1±1.0	1.0±1.0	0.4±0.2
Deferribacteres	0.1±0.1	0.4±0.3	0.2±0.2
Actinobacteria	0.1±0.0	0.2±0.1	0.2±0.2
Cyanobacteria	0.3±0.2	0.1±0.1#	0.5±0.4*
Saccharibacteria_TM7	0.0±0.0	0.0±0.0	0.0±0.0

Values indicate means±SD. $^{\#}p < 0.05$ vs. NC. $^{*}p<0.05$ vs. EV.

Table S7. Effects of NKc on the gut microbiota composition at the family level in mice with pEVs-induced periodontitis and cognitive impairment

Taxon Name	Composition (%)		
	NC	EV	Mix
Muribaculaceae	34.6±9.5	29.4±9.5	28.4±8.3
Lachnospiraceae	25.5±7.0	30.1±9.4	33.5±2.8
Prevotellaceae	14.6±4.1	9.4±3.9#	10.9±3.3
Ruminococcaceae	6.5±1.0	8.0±2.6	8.3±1.6
Lactobacillaceae	4.8±2.7	5.5±4.0	2.6±1.8
Helicobacteraceae	3.5±2.4	5.4±3.2	5.0±3.7
Bacteroidaceae	3.2±2.8	2.8±1.3	3.3±1.9
Rikenellaceae	1.7±0.8	1.9±1.1	2.3±1.9
Desulfovibrionaceae	1.3±0.4	2.1±0.9	2.1±0.8
Christensenellaceae	0.7±1.0	0.5±0.5	0.3±0.2
Porphyromonadaceae	0.6±0.4	0.4±0.2	0.4±0.2
Mycoplasmataceae	0.6±0.4	0.8±0.9	0.2±0.1
AC160630_f	0.4±0.2	0.3±0.2	0.3±0.2
FR888536_f	0.3±0.2	0.1±0.1#	0.5±0.4 *
Dehalobacterium_f	0.2±0.1	0.3±0.1	0.2±0.1
Odoribacteraceae	0.2±0.2	0.2±0.1	0.3±0.3

PAC000197_f	0.2±0.3	0.1±0.1	0.1±0.1
Acholeplasmataceae	0.2±0.4	0.0±0.0	0.0±0.0
Sutterellaceae	0.2±0.1	0.2±0.4	0.2±0.4
Erysipelotrichaceae	0.1±0.1	0.1±0.2	0.1±0.1
Deferribacteraceae	0.1±0.1	0.4±0.3	0.2±0.2
Rhodospirillaceae	0.1±0.1	0.1±0.2	0.2±0.1
Clostridiaceae	0.1±0.1	0.1±0.1	0.1±0.1
Coriobacteriaceae	0.1±0.0	0.1±0.1	0.1±0.1
Mogibacterium_f	0.1±0.0	0.1±0.0	0.1±0.0

Values indicate means±SD. $^{\#}p < 0.05$ vs. NC. $^{*}p<0.05$ vs. EV.

Table S8. Effects of NKc on the gut microbiota composition at the genus level in mice with pEVs-induced periodontitis and cognitive impairment

Taxon Name	Composition (%)		
	NC	EV	Mix
Prevotellaceae_uc	10.6±2.4	2.8±1.7#	5.6±2.7
PAC000186_g	10.3±6.0	4.8±1.4	5.1±1.8
PAC001068_g	7.9±1.6	8.0±2.1	8.1±2.9
Lactobacillus	4.8±2.6	5.5±3.9	2.6±1.8
PAC000198_g	4.3±1.2	3.3±1.7	3.1±1.5
PAC001512_g	3.7±1.0	2.0±1.0#	1.5±1.2
KE159538_g	3.5±2.7	2.6±2.1	2.7±1.6
Helicobacter	3.5±2.4	5.4±3.2	4.9±3.7
PAC000664_g	3.4±3.6	3.1±3.7	3.4±3.4
Bacteroides	3.2±2.8	2.8±1.3	3.3±1.9
Prevotella	2.0±1.2	2.2±1.4	2.5±1.3
PAC001112_g	1.9±2.2	3.1±5.6	3.2±5.2
PAC001091_g	1.8±3.1	0.3±0.3	0.7±1.1
Oscillibacter	1.7±0.6	2.2±1.1	2.2±0.4
Pseudoflavonifractor	1.6±0.5	2.4±0.8	2.2±0.5
PAC001074_g	1.4±1.4	2.4±1.2	1.6±1.0
Muribaculum	1.4±0.5	1.6±1.5	1.6±1.5
PAC001127_g	1.2±0.5	0.8±0.6	1.3±0.4
LLKB_g	1.2±1.0	1.9±0.9	1.9±1.7
Alistipes	1.2±0.8	1.4±0.8	1.9±1.8
Alloprevotella	1.1±2.5	0.4±0.3	0.7±0.7
PAC001092_g	1.0±1.0	1.8±1.7	1.9±1.5
PAC001105_g	1.0±0.6	2.3±2.8	1.7±1.2
LT706945_g	1.0±0.2	1.5±0.7	1.5±0.5

PAC001124_g	1.0±0.6	0.4±0.4	1.7±1.6

Values indicate means±SD. #p < 0.05 vs. NC. *p<0.05 vs. EV.

Table S9. Effects of NKc on the gut microbiota composition at the species level in mice with pEVs-induced periodontitis and cognitive impairment

Taxon Name	Composition (%)		
	NC	EV	Mix
PAC002447_s	6.0±5.1	0.2±0.3#	0.2±0.1
EF097112_s	3.8±1.3	3.6±1.9	3.7±1.8
EU791023_s	3.7±1.0	1.9±1.0#	1.5±1.2
PAC000198_s	3.0±1.1	1.4±0.9#	1.1±1.1
PAC001065_s group	2.2±0.8	3.7±1.2#	3.8±1.5
Helicobacter rodentium group	2.1±1.7	3.9±3.5	3.8±4.1
PAC001064_s	1.8±1.0	0.7±0.4#	0.8±0.5
Lactobacillus murinus group	1.6±1.8	2.9±2.0	1.0±0.7
PAC001558_s	1.6±3.1	0.2±0.2	0.1±0.1
EU622763_s group	1.5±1.4	1.7±1.7	1.5±1.8
PAC001072_s	1.4±0.6	0.9±0.3	1.2±0.6
KE159538_s	1.4±1.7	0.7±0.4	0.6±0.1
Lactobacillus reuteri group	1.4±0.6	1.1±0.9	0.8±0.6
AB606242_s	1.3±2.9	0.1±0.1	0.6±0.8
AB599946_s	1.3±1.0	1.4±0.9	2.0±1.6
Helicobacter japonicus	1.3±1.2	1.3±1.1	1.0±0.7
PAC002399_s	1.2±0.5	0.8±0.6	1.2±0.4
PAC001070_s group	1.2±0.6	1.3±1.3	2.1±2.4
Lactobacillus gasseri group	1.1±0.5	0.8±0.5	0.5±0.2
PAC002479_s	1.1±2.5	0.4±0.3	0.7±0.7
KE159538_g_uc	1.1±2.5	0.0±0.0	0.0±0.0
Bacteroides acidifaciens group	1.0±1.0	0.9±0.3	0.9±0.6
PAC002478_s	1.0±0.2	1.4±0.7	1.4±0.5
PAC001074_s	0.9±0.7	2.4±1.2#	1.5±1.0
FJ880724_s	0.9±1.5	3.9±2.4#	2.1±3.1

Values indicate means±SD. #p < 0.05 vs. NC. *p<0.05 vs. EV.

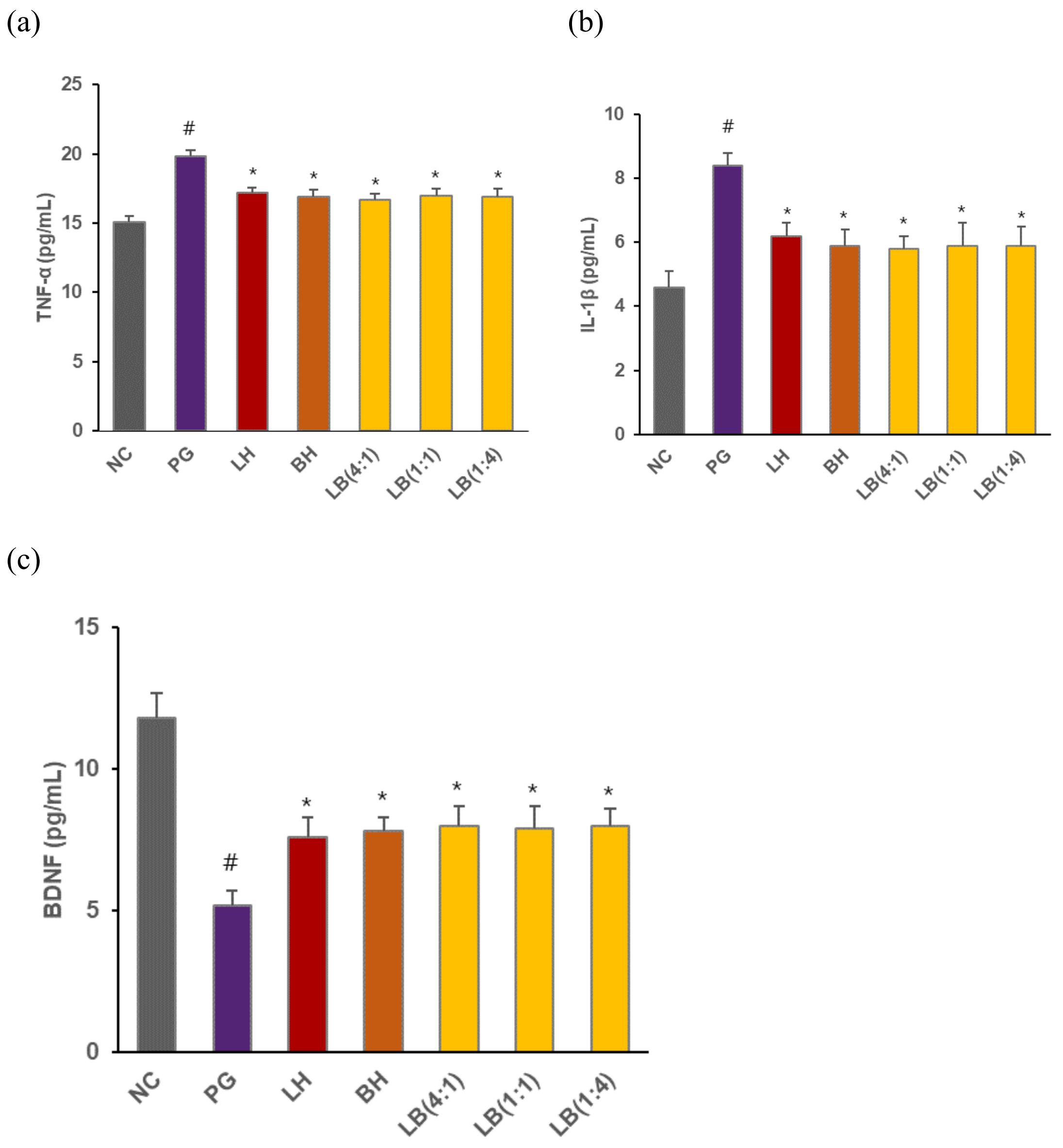

Figure S1. The combined effects of NK357 and NK391 on the TNF-α and IL-1β expression in *P. gingivalis*-stimulated BV2 cells and increased BDNF expression in *P. gingivalis*-stimulated SH-SY5Y cells. Effects on *P. gingivalis*-induced TNF-α (a) and IL-1β expression (b) in BV2 cells. (c) Effects on *P. gingivalis*-suppressed BDNF expression in SH-5Y cells. NC, treated with vehicle; PG, treated with 1×10^5 CFU/mL of *P. gingivalis*; LL, 1×10^5 CFU/mL of NK357 with PG; LH, 1×10^5 CFU/mL of NK357 with PG; BH, 1×10^5 CFU/mL of NK391 with PG; LB(4:1), treated with 1×10^5 CFU/mL of NK357 and NK391 (4:1) mix; LB(1:1), treated with 1×10^5 CFU/mL of NK357 and NK391 (1:1) mix; LB(1:4), treated with 1×10^5 CFU/mL of NK357 and NK391 (1:4) mix. Data were described as mean ± SD (n = 4). $^{\#}p<0.05$ vs. NC. $^{*}p<0.05$ vs. PG.
